# Supplementary material for: Early Neonatal Meconium Does Not Have a Demonstrable Microbiota Determined through Use of Robust Negative Controls with cpn60-Based Microbiome Profiling
Source: Microbiol Spectr. 2021 Sep 29;9(2):e00067-21. doi: 10.1128/Spectrum.00067-21 (PMC8557823; doi:10.1128/Spectrum.00067-21)
Supplement: SUPPLEMENTAL FILE 2 — Supplemental material. Download SPECTRUM00067-21_Supp_2_seq8.pdf, PDF file, 0.6 MB [file spectrum00067-21_supp_2_seq8.pdf]

Supplementary Table S1: Primers used in this study (**I** = inosine; **Y** = C/T; **K** = G/T; **R** = A/G; **S** = G/C).

Target	Forward primer	Reverse primer
*cpn*60 (containing Illumina index primer landing sites; underlined)	**M279:** 5’-TCG TCG GCA GCG TCA GAT GTG TAT AAG AGA CAG GAI III GCI GGI GAY GGI ACI ACI AC-3’	**M280:** 5’-GCT TCG TGG GCT CGG AGA TGT GTA TAA GAG ACA GYK IYK ITC ICC RAA ICC IGG IGC YTT-3’
	**M1612:** 5’-TCG TCG GCA GCG TCA GAT GTG TAT AAG AGA CAG GAI III GCI GGY GAC GGY ACS ACS AC-3’	**M1613:** 5’-GCT TCG TGG GCT CGG AGA TGT GTA TAA GAG ACA GCG RCG RTC RCC GAA GCC SGG IGC CTT-3’
Human mitochondrial cytochrome c oxidase I	**JH0241:** 5’-CAC CTT CTT CGA CCC CGC CG-3’	**JH0242:** 5’-TGC TTC CGT GGA GTG TGG CG-3’
16S rRNA gene (qPCR)	**SRV3-1:** 5’-CGG YCC AGA CTC CTA C-3’	**SRV3-2:** 5’-TTA CCG CGG CTG CTG GCA C-3’
cpn60 (containing M13 sequencing primer landing sites; underlined)	**H729:** 5'-CGC CAG GGT TTT CCC AGT CAC GAC GAI III GCI GGI GAY GGI ACI ACI AC-3'	**H730:** 5'-AGC GGA TAA CAA TTT CAC ACA GGA YKI YKI TCI CCR AAI CCI GGI GCY TT-3'

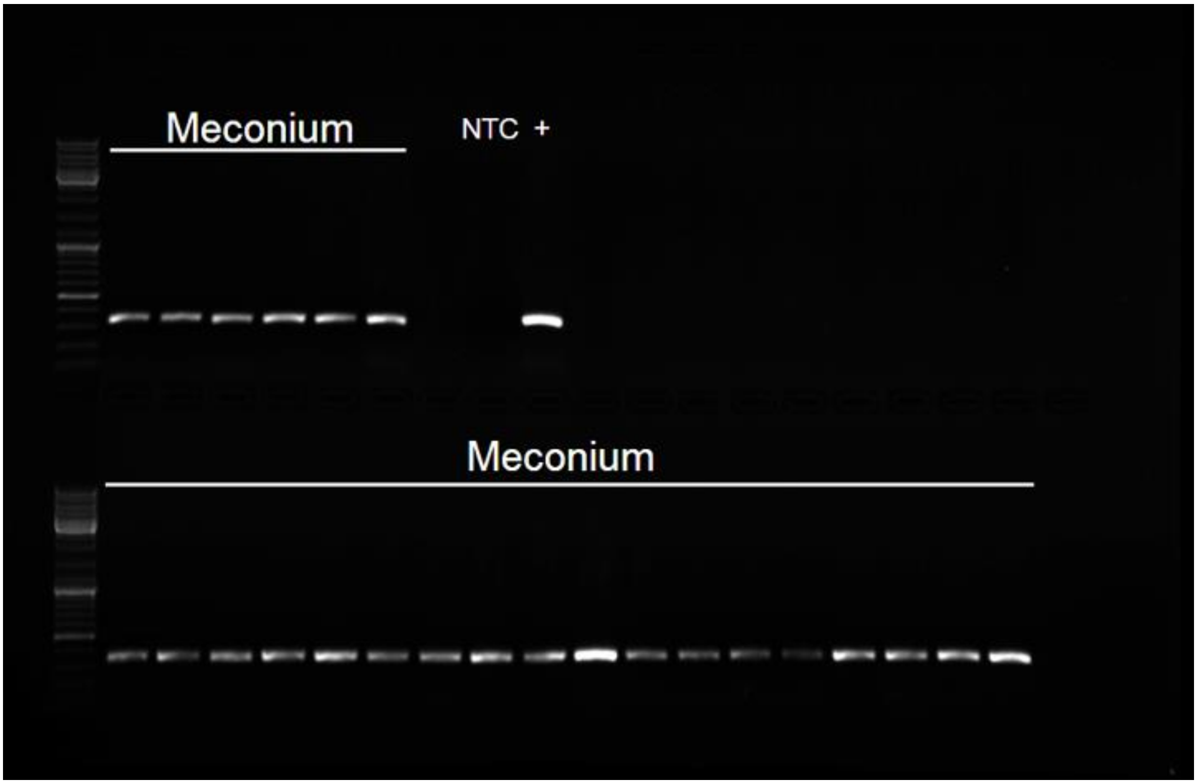

Supplementary Figure S1 All meconium samples contain amplifiable DNA: (**A**) Representative ethidium bromide gel confirming amplification of the human mitochondrial COX-1 gene from meconium samples. All samples were PCR-positive for COX-1, ruling out PCR inhibition as a cause of low read numbers observed in meconium microbiome profiles (NTC = no-template control; + = positive control, total genomic DNA extracted from human vaginal swab fluid).

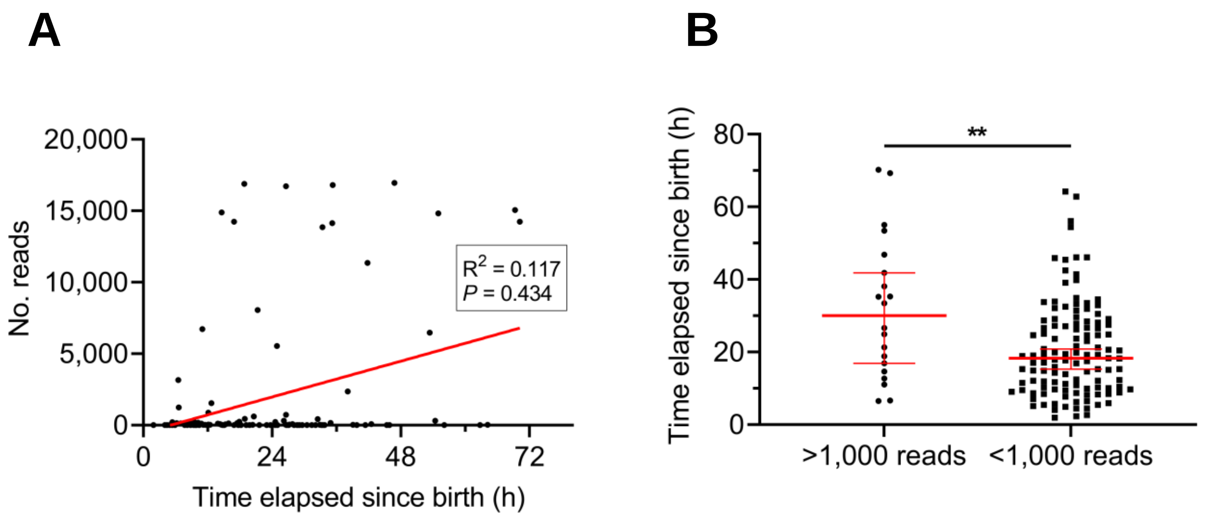

Supplementary Figure S2 Longer intervals from birth to sample collection in meconium samples with higher read numbers: (A) Number of hours elapsed from birth to sample collection is plotted against read counts for 136 meconium samples. Time since birth showed a weakly positive but non-significant correlation with read numbers (R^2 = 0.117; Spearman's rank, P = 0.434, ρ = 0.096). (B) Number of hours elapsed from birth to collection time for all meconium samples. Mean number of hours elapsed since birth was significantly higher for samples with >1,000 reads (Mann-Whitney U test, P <0.01, U = 766.5). Red line indicates median (±95% CI).

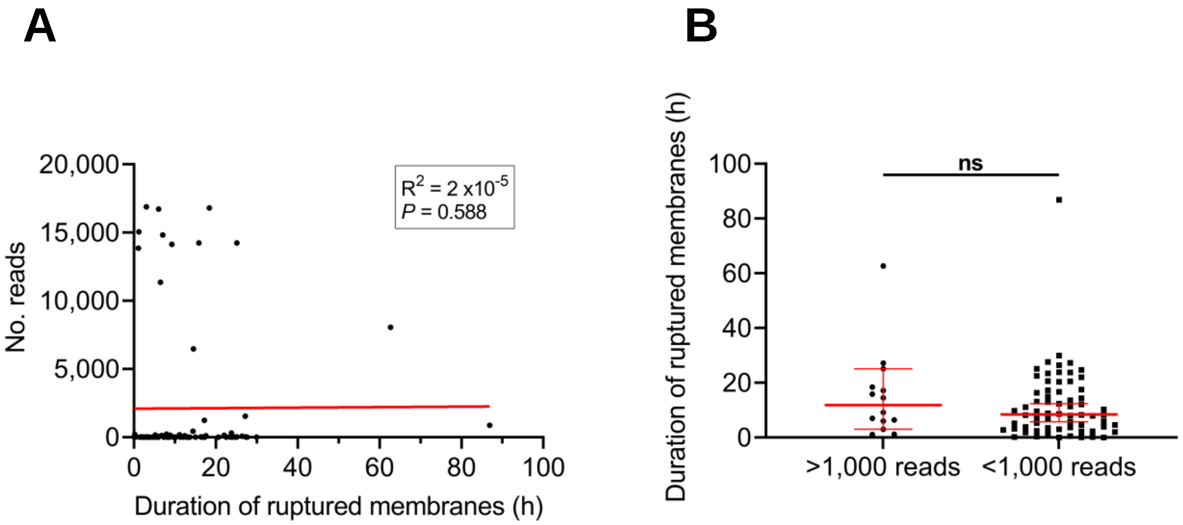

Supplementary Figure S3 No difference in duration of ruptured membranes in meconium samples with higher read numbers: (A) Number of hours elapsed from membrane rupture to birth is plotted against read counts for meconium samples obtained from infants delivered vaginally or by emergent caesarean (n = 80). Duration of ruptured membranes showed no significant correlation with read numbers (R^2 = 2 x10-5; Spearman's rank, P = 0.588, ρ = 0.061). (B) Number of hours elapsed from membrane rupture to birth for all meconium samples grouped by read numbers. Mean number of hours between membrane rupture and birth was not significantly different (Mann-Whitney U test, P = 0.454, U = 402). Red line indicates median (±95% CI).

Supplementary Table S2: Read count summaries for the most common nearest neighbours found in at least 4 samples or controls across the entire dataset. Note: % similarity denotes the range of nucleotide similarities of all ASVs collapsed into a given nearest neighbour to the best match in cpnDB (* = single ASV).

Nearest neighbour (NN)	# Samples (control)	# Reads (control)			% Similarity
		Total	Median	Range	
Hyphomicrobium zavarzinii	24<br>(3)	397<br>(156)	12<br>(57)	107 - 2<br>(77 - 22)	80.6 - 80.2
Shigella sonnei	18<br>(4)	81,037<br>(449)	12<br>(89)	16961 - 1<br>(271 - 1)	100 - 98.4
Bradyrhizobium arachidis	14<br>(2)	149<br>(6)	7<br>(3)	44 - 2<br>(3)	90.0 - 89.6
Chelatococcus spp. GW1	11<br>(1)	137<br>(2)	10<br>(2)	26 - 1<br>(2)	77.7 - 76.9
Lactobacillus crispatus	8<br>(2)	172<br>(17)	3<br>(9)	143 - 1<br>(15 - 2)	100 - 99.6
Enterococcus faecalis	8<br>(0)	2,239<br>(0)	130<br>(n/a)	1,453 - 3<br>(n/a)	99.6 - 99.2
Ralstonia insidiosa	8<br>(0)	120<br>(0)	10<br>(n/a)	39 - 2<br>(n/a)	99.2 - 98.4
Parabacteroides spp. D25	7<br>(0)	19,373<br>(0)	60<br>(n/a)	11,854 - 2<br>(n/a)	100 - 99.2
Escherichia coli	6<br>(0)	38,537<br>(0)	5,710<br>(n/a)	14832 - 12<br>(n/a)	99.2 - 98.4
Lactobacillus gasseri	5<br>(0)	652<br>(0)	7<br>(n/a)	420 - 1<br>(n/a)	100 - 97.2
Bacteroides vulgatus	5<br>(0)	18,826<br>(0)	1,130<br>(n/a)	11,031 - 7<br>(n/a)	100 - 99.6
Propionibacterium spp. 434-HC2	4<br>(1)	40<br>(7)	11<br>(7)	15 - 3<br>(7)	100 - 98.8
Shigella flexneri	5<br>(0)	13,241<br>(0)	2<br>(0)	13,138 - 1<br>(n/a)	99.6 - 99.2
Moraxella osloensis	5<br>(0)	361<br>(0)	29<br>(n/a)	194 - 2<br>(n/a)	97.6 - 96.4
Pseudomonas lactis	5<br>(0)	259<br>(0)	24<br>(n/a)	147 - 6<br>(n/a)	96.4 - 96.0
Streptococcus mitis	4<br>(0)	359<br>(0)	25<br>(n/a)	306 - 3<br>(n/a)	100 - 92.8
Rothia mucilaginosa	4<br>(0)	7,173<br>(0)	1,207<br>(n/a)	4,736 - 24<br>(n/a)	98.8 - 85.9
Lysobacter spongiicola	4<br>(0)	124<br>(0)	28<br>(n/a)	62 - 6<br>(n/a)	* 89.9
Arthrobacter nitrophenolicus	3<br>(1)	79<br>(5)	22<br>(5)	54 - 3<br>(5)	* 78.7

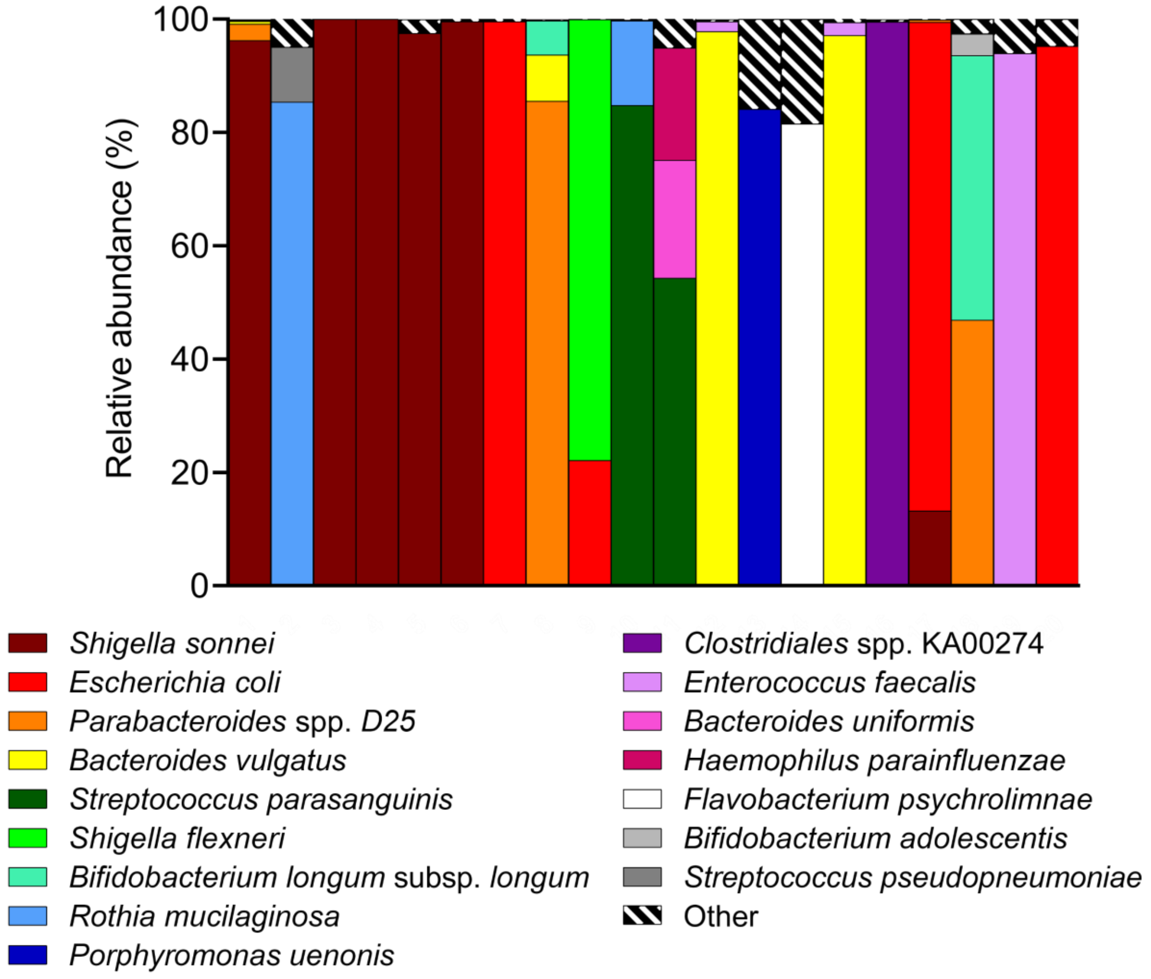

Supplementary Figure S4 High read number meconium samples are comprised of common enteric species often found in stool: Microbiome profiles of the 20 meconium samples yielding >1,000 *cpn*60 reads, expressed as relative abundance. All but one of these samples are dominated by a single NN, demonstrating the simplicity of the neonatal microbiome compared to that of older infants and adults. Common bacterial species known to inhabit the gastrointestinal tract of humans, such as *E. coli* / *Shigella* spp., *Bacteroides vulgatus* and *Streptococcus* spp. are abundant across this subset of samples.

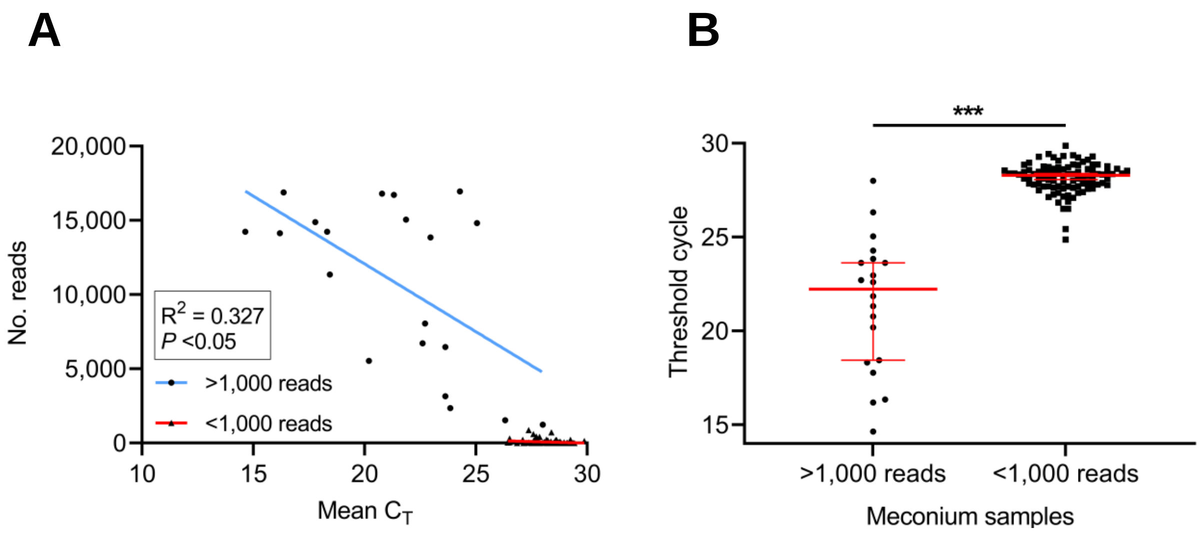

Supplementary Figure S5 Read numbers and 16S rRNA qPCR cycle thresholds are well-correlated: (A) Mean 16S rRNA qPCR threshold cycles (CT; from duplicate reactions) plotted against number of reads for 114 samples with both data available. CT values of samples with >1,000 reads show a significant inverse correlation with read number (R2 = 0.327; Spearman's rank, P <0.05, ρ = -0.447) while those with <1,000 reads do not (R2 = 0.035; Spearman's rank, P = 0.252, , ρ = -0.116). (B) Meconium samples with >1,000 reads had a significantly lower threshold cycle than those with <1,000 reads (Mann-Whitney U test, P <0.01, U = 38.5). Red line indicates median (±95% CI).

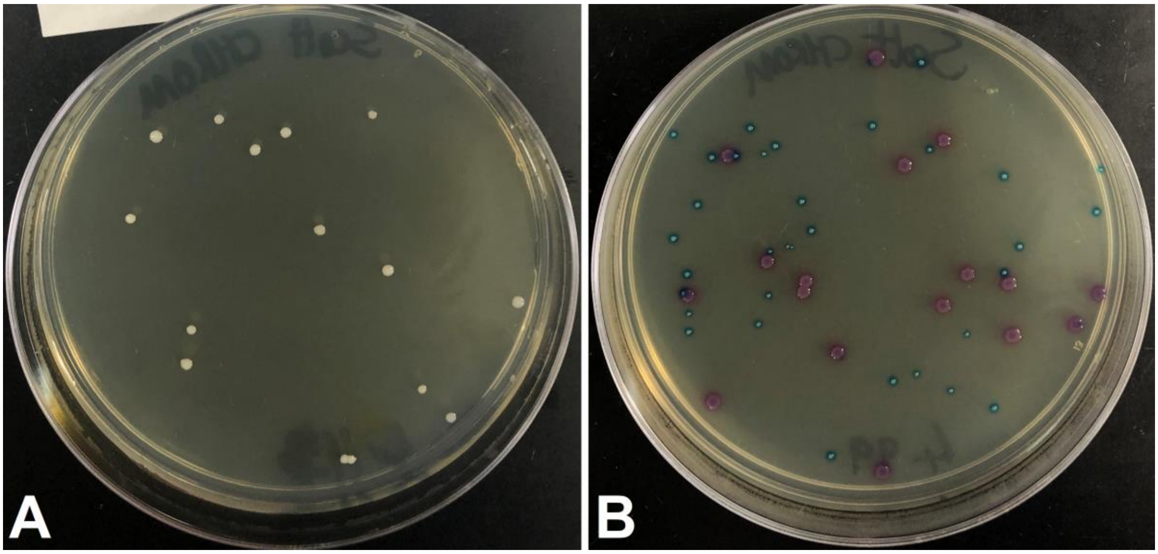

Supplementary Figure S6 Undiluted meconium cultures yield very few colonies: Two representative plates of CHROM Orientation agar inoculated with meconium (resuspended in PBS <u>without serial dilution</u>) incubated at 37°C overnight under aerobic conditions. Plates are representative of the entire dataset and exhibit a small number of colonies of (**A**) *Staphylococcus epidermidis*, (**B**) *Escherichia coli* (purple) and *Enterococcus faecalis* (blue).

Supplementary Table S3 ***Escherichia coli*** **PFGE similarity matrix:** Dice similarity coefficients for the 12 *Escherichia coli* isolates cultured from meconium, used to construct the dendrogram in Figure 3A. The colour of coefficients indicates strain relatedness: >90 = indistinguishable (I), >80 = related (R), <80 unrelated (U).

	94	521	129	195	251	385	262	499	231	412	141	293
**94**	-											
**521**	93.8	-										
**129**	66.7	70.6	-									
**195**	62.9	60.6	54.1	-								
**251**	74.3	66.7	75.7	83.3	-							
**385**	61.1	47.1	63.2	70.3	64.9	-						
**262**	43.8	40.0	53.0	54.6	54.6	70.6	-					
**499**	59.1	52.4	60.9	62.2	62.2	69.6	61.9	-				
**231**	54.6	58.1	51.4	47.1	41.2	51.4	51.6	55.8	-			
**412**	44.5	47.1	47.4	59.5	48.7	57.9	64.7	60.9	79.8	-		
**141**	50.0	46.7	64.7	60.6	54.6	53.0	46.7	52.4	64.5	53.0	-	
**293**	48.3	44.5	58.1	46.7	66.7	51.6	44.5	46.2	35.7	38.7	44.5	-

**>90**	**I**
**>80**	**R**
**<80**	**U**

Supplementary Table S4 *Staphylococcus epidermidis* PFGE similarity matrix: Dice similarity coefficients for the 25 *Staphylococcus epidermidis* isolates cultured from meconium, used to construct the dendrogram in Figure 3B. The colour of coefficients indicates strain relatedness: >90 = indistinguishable (I), >80 = related (R), <80 unrelated (U).

>90	I
>80	R
<80	U

	398 P	495 P	318 P	227 W	297 P	483 P	471 W	495 W	239 W	298 P	191 W	390 W	364 W	330 P	94 W	113 Y	53 P	94 W	67 P	281 W	265 W	10 P	81 P	129 W	479 P
**398 P**	-																								
**495 P**	78.6	-																							
**318 P**	60.0	73.3	-																						
**227 W**	76.9	53.9	57.2	-																					
**297 P**	69.0	55.2	71.0	79.5	-																				
**483 P**	68.8	62.5	64.7	66.7	72.7	-																			
**471 W**	66.7	66.7	68.8	71.4	71.0	70.6	-																		
**495 W**	69.2	53.9	64.3	66.7	66.7	66.7	71.4	-																	
**239 W**	66.7	53.3	56.3	57.2	64.5	64.7	68.8	71.4	-																
**298 P**	77.4	64.5	60.6	69.0	81.3	68.6	60.6	62.1	78.8	-															
**191 W**	64.3	57.2	60.0	38.5	55.2	50.0	60.0	46.2	48.3	66.7	-														
**390 W**	61.5	61.5	57.2	50.0	66.7	66.7	57.2	50.0	57.2	48.3	23.1	-													
**364 W**	59.3	29.6	41.4	72.0	71.4	51.6	48.3	56.0	62.1	66.7	44.5	72.0	-												
**330 P**	53.9	53.9	42.9	41.7	51.9	66.7	35.7	33.3	57.2	62.1	38.5	66.7	64.0	-											
**94 W**	34.5	55.2	51.6	51.9	46.7	66.7	45.2	51.9	45.2	50.0	34.5	37.0	50.0	44.5	-										
**113 Y**	66.7	37.0	55.2	64.0	42.9	45.2	55.2	48.0	41.4	66.7	37.0	40.0	61.5	40.0	64.3	-									
**53 P**	53.3	66.7	62.5	50.0	38.7	53.0	62.5	57.2	50.0	42.4	48.3	35.7	20.7	35.7	38.7	41.4	-								
**94 Y**	50.0	57.2	46.7	53.9	48.3	62.5	60.0	46.2	33.3	58.1	50.0	53.9	44.5	46.2	55.2	51.9	66.7	-							
**67 P**	55.2	48.3	51.6	66.7	60.0	54.6	45.2	44.5	51.6	62.5	48.3	37.0	35.7	44.5	53.3	50.0	71.0	69.0	-						
**281 W**	51.9	51.9	48.3	64.0	42.9	45.2	55.2	64.0	48.3	40.0	44.5	40.0	46.2	16.0	35.7	38.5	62.1	59.3	71.4	-					
**265 W**	48.0	56.0	44.5	43.5	46.2	55.2	44.5	60.9	37.0	42.9	48.0	43.5	41.7	34.8	38.5	41.7	59.3	64.0	53.9	66.7	-				
**10 P**	56.0	56.0	37.0	43.5	38.5	48.3	44.5	52.2	59.3	57.2	40.0	34.8	41.7	17.4	38.5	41.7	44.5	48.0	53.9	41.7	63.6	-			
**81 P**	38.5	53.9	57.2	41.7	59.3	53.3	42.9	41.7	50.0	55.2	61.5	50.0	56.0	25.0	66.7	40.0	42.9	53.9	59.3	56.0	52.2	69.6	-		
**129 W**	59.3	44.5	41.4	48.0	35.7	38.7	48.3	64.0	48.3	53.3	59.3	40.0	38.5	24.0	35.7	38.5	55.2	37.0	57.2	53.9	50.0	66.7	56.0	-	
**479 P**	35.7	28.6	33.3	30.8	41.4	31.3	13.3	30.8	33.3	58.1	35.7	23.1	29.6	30.8	41.4	37.0	40.0	50.0	48.3	51.9	32.0	40.0	46.2	44.5	-

Supplementary Table S5 *Enterococcus faecalis* PFGE similarity matrix: Dice similarity coefficients for the 24 *Enterococcus faecalis* isolates cultured from meconium, used to construct the dendrogram in Figure 3C. The colour of coefficients indicates strain relatedness: >90 = indistinguishable (I), >80 = related (R), <80 unrelated (U).

	277	364	398	405	235	352	239	499	510	37	141	187	223	349	366	14	30	81	265	402	412	514	536	18
**277**	-																							
**364**	60.6	-																						
**398**	53.7	63.2	-																					
**405**	57.2	56.3	70.0	-																				
**235**	48.7	58.8	57.2	61.1	-																			
**352**	40.0	43.8	40.0	35.3	77.8	-																		
**239**	48.5	40.0	57.9	37.5	76.5	56.3	-																	
**499**	60.6	60.0	52.6	37.5	70.6	75.0	66.7	-																
**510**	48.5	66.7	52.6	50.0	64.7	62.5	60.0	93.3	-															
**37**	60.6	60.0	52.6	50.0	58.8	50.0	46.7	80.0	80.0	-														
**141**	53.0	58.1	61.5	54.6	62.9	60.6	51.6	90.3	71.0	96.8	-													
**187**	50.0	60.6	53.7	62.9	64.9	62.9	60.6	72.7	72.7	66.7	76.5	-												
**223**	44.5	66.7	53.7	62.9	70.3	68.6	66.7	66.7	66.7	66.7	64.7	100.0	-											
**349**	40.0	50.0	55.0	58.8	77.8	64.7	62.5	81.3	75.0	62.5	60.6	91.4	97.2	-										
**366**	36.4	46.7	57.9	50.0	70.6	62.5	40.0	73.3	73.3	73.3	71.0	90.9	90.9	93.8	-									
**14**	55.6	42.4	58.5	62.9	59.5	57.2	60.6	60.6	66.7	78.8	70.6	66.7	72.2	62.9	54.6	-								
**30**	55.6	42.4	58.5	57.2	54.1	51.4	60.6	66.7	66.7	72.7	70.6	66.7	61.1	51.4	48.5	100.0	-							
**81**	63.2	45.7	55.8	64.9	61.5	43.3	62.9	57.2	57.2	68.6	72.2	63.2	42.1	54.1	62.9	94.7	94.7	-						
**265**	64.9	58.8	57.2	50.0	63.2	61.1	76.5	58.8	64.7	47.1	57.2	59.5	59.5	55.6	47.1	70.3	86.5	82.1	-					
**402**	51.4	43.8	45.0	47.1	61.1	53.0	62.5	62.5	62.5	62.5	66.7	62.9	68.6	53.0	62.5	68.6	68.6	70.3	66.7	-				
**412**	57.2	56.3	35.0	41.2	38.9	53.0	43.8	56.3	50.0	56.3	54.6	57.2	57.2	53.0	50.0	57.2	57.2	59.5	50.0	35.3	-			
**514**	54.6	60.0	57.9	50.0	35.3	56.3	40.0	60.0	66.7	53.3	58.1	60.6	48.5	56.3	73.3	42.4	42.4	51.4	41.2	56.3	75.0	-		
**536**	48.5	60.0	52.6	50.0	35.3	50.0	33.3	40.0	53.3	46.7	51.6	48.5	42.4	43.8	53.3	42.4	42.4	45.7	35.3	37.5	62.5	73.3	-	
**18**	40.0	37.5	45.0	53.0	61.1	64.7	43.8	50.0	43.8	50.0	42.4	62.9	62.9	53.0	43.8	45.7	45.7	43.3	50.0	47.1	47.1	50.0	37.5	-

**>90**	**I**
**>80**	**R**
**<80**	**U**

Supplementary Table S6: Birth months and delivery modes for infants from whose meconium clonal isolates of *Enterococcus faecalis* were cultured. Infants in each clonal group were born at similar times, providing a potential explanation for the isolation of identical *Enterococcus* strains.

Clonal Group	Sample ID	Birth month	Delivery mode
1	0037	Apr '18	Vaginal
	0141	May '18	Vaginal
2	0499	Aug '18	Vaginal
	0510	Sep '18	Vaginal
3	0187	Jun '18	Vaginal
	0223	Jun '18	C/S elective
	0366	Aug '18	C/S elective
	0349	Aug '18	Vaginal
4	0014	Apr '18	C/S emergency
	0030	Apr '18	C/S elective
	0081	Apr '18	C/S elective
	0265	Jul '18	C/S emergency
